# 

**Published:** 1886-12-04

**Authors:** 


### THE HOSPITAL

An Institution, Family, and Parochial Journal of
Hospitals, Asylums, & all Agencies for the Care of the Sick, Criticism & News.

### CONTENTS.

**The Golden Age of Pauperism** - - - 155

**Words of Consolation.**—X. Cheerfulness - 156

**Poetry.**—In the Night Time - - - 156

**The Story of the Hospitals.**—The London Hospital—*continued* - - - - - 157

**Harvey Interviewed.**—By Our Special Commissioner - - - - - - - 158

**Sister Olive:** A Hospital Romance.—By W. J. Eccott 159

**The Editor's Letter Box.**—The General Hospital, Nottingham - - - - - - 161

**Total Abstinence and Common Sense** - - 161

**Special Notices** - - - - - - - 162

**Notes and News.**—H.R.H. Princess Christian and the After Care Association.—Bequests.—Miscellaneous.—Brompton Hospital and Trained Nurses.—Vacancies.—The Guest Hospital at Dudley.—Jubilee Cottage Hospital for Melton.—The Driffield Cottage Hospital.—Concert at the Wrexham Infirmary.—Legacies to the Brompton Consumption Hospital.—Improvements at the Chichester Infirmary.—Hospital and Infirmary Reports, etc. - - - - - - - - - 162

**Annotations.**—The Analysts on their Defence.—Flogging in Board Schools.—Hydrophobia: Society for its Prevention - - - - - - - 165

**The "Truth" Toy Fund** - - - - - 166

**Reminiscences of the Insane.**—By a Mental Physician - - - - - - - - 167

**Till the Doctor Comes.**—General Hints on Nursing 168

**The Training of Midwives** - - - - 169

**In- and Out-Patients' Hospital Diary** - - 170

**The Children's Corner.**—Puzzles, Answers, etc. - vi

**The Hospital Charity Box.**—Prizes and Results - vi

